# Dysbiosis of gut microbiota in inflammatory bowel disease: Current therapies and potential for microbiota-modulating therapeutic approaches

**DOI:** 10.17305/bjbms.2020.5016

**Published:** 2021-06

**Authors:** Dikhnah Alshehri, Omar Saadah, Mahmoud Mosli, Sherif Edris, Rashad Alhindi, Ahmed Bahieldin

**Affiliations:** 1 Department of Biological Sciences, Faculty of Science, King Abdulaziz University, Jeddah, Saudi Arabia; 2 Department of Biology, Faculty of Science, Tabuk University, Tabuk, Saudi Arabia; 3 Department of Pediatrics, Faculty of Medicine, King Abdulaziz University, Jeddah, Saudi Arabia; 4 Inflammatory Bowel Disease Research Group, King Abdulaziz University, Jeddah, Saudi Arabia; 5 Department of Medicine, Faculty of Medicine, King Abdulaziz University, Jeddah, Saudi Arabia; 6 Department of Genetics, Faculty of Agriculture, Ain Shams University, Cairo, Egypt; 7 Princess Al Jawhara Albrahim Center of Excellence in Research of Hereditary Disorders (PACER-HD), Faculty of Medicine, King Abdulaziz University, Jeddah, Saudi Arabia

**Keywords:** Inflammatory bowel disease, microbiota, dysbiosis, anti-TNF-α, microbiome-modulating therapy

## Abstract

There is a growing body of evidence reinforcing the unique connections between the host microbiome, health, and diseases. Due to the extreme importance of the symbiotic relationship between the intestinal microbiome and the host, it is not surprising that any alteration in the gut microbiota would result in various diseases, including inflammatory bowel disease (IBD), Crohn’s disease (CD) and ulcerative colitis (UC). IBD is a chronic, relapsing-remitting condition that is associated with significant morbidity, mortality, compromised quality of life, and costly medical care. Dysbiosis is believed to exacerbate the progression of IBD. One of the currently used treatments for IBD are anti-tumor necrosis factor (TNF) drugs, representing a biologic therapy that is reported to have an impact on the gut microbiota composition. The efficacy of anti-TNF agents is hindered by the possibility of non-response, which occurs in 10-20% of treated patients, and secondary loss of response, which occurs in up to 30% of treated patients. This underscores the need for novel therapies and studies that evaluate the role of the gut microbiota in these conditions. The success of any therapeutic strategy for IBD depends on our understanding of the interactions that occur between the gut microbiota and the host. In this review, the health and disease IBD-associated microbiota patterns will be discussed, in addition to the effect of currently used therapies for IBD on the gut microbiota composition, as well as new therapeutic approaches that can be used to overcome the current treatment constraints.

## INTRODUCTION

It has been estimated that more than one million residents of the United States and 2.5 million individuals living in the European Union suffer from inflammatory bowel disease (IBD). IBD is a chronic inflammatory disorder that has been associated with high morbidity and mortality, low quality of life, and financially demanding medical care [1,2]. The incidence of IBD has been rapidly increasing in developed and industrialized countries over the last two decades. Furthermore, IBD is an inflammatory disease of the gastrointestinal (GI) tract that exhibits a chronic relapsing-remitting course. The causes of this disease are multi-factorial, but it can be subdivided into two main types, Crohn’s disease (CD) and ulcerative colitis (UC). Even though both conditions have similar clinical and pathological presentations, it seems that the main biological processes involved in the development of CD and UC are different [3]. CD can affect any part of the GI tract with a particular preference for the terminal ileum, whereas UC can only affect the large intestine, i.e., the colon [4]. In a minority of cases where only the colon is affected, CD can be indistinguishable from UC, and such cases are often described as “indeterminate colitis.” Both conditions can cause irreversible impairment of the structure and function of the GI tract [3].

The diversity and composition of the human gut microbiota are believed to play a critical role in human health and the development of disease. A well-balanced composition of microbes in the gut (symbiosis) is recognized as crucial for maintaining a normal and healthy GI tract. Gut microbes have also been implicated in the pathophysiology of inflammation, especially in patients with confirmed IBD [5]. However, although the global alterations in the gut microbial ­communities of patients with IBD have been recognized and the research so far has found associations between microbial factors and inflammation, it is important to emphasize that there are still no clear conclusions to be drawn [6].

**FIGURE 1:**
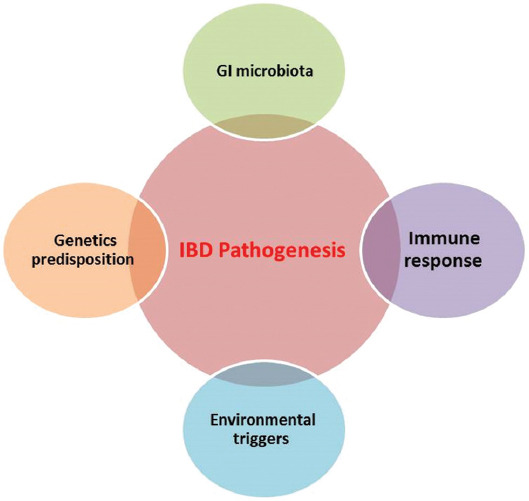
Interaction of various factors contributing to chronic intestinal inflammation in a genetically susceptible host, modified from Sartor [56]

The exact pathogenesis of IBD remains unclear, but it has been noted that IBD occurs as a result of complicated interactions between genetic predisposition, environmental factors (diet, antimicrobial usage, smoking, etc.), socio-economic development, and microbial colonization [7]. One of the most common causes for the development of IBD is the inappropriate perpetuation of innate and adaptive immune factors in response to environmental triggers. This excessive immune response causes disregulation of cytokines and chronic inflammation (Figure 1) [6,8].

In this review, we explored the microbiota patterns associated with healthy and IBD-affected intestines and the effect of current IBD therapies on the gut microbiota composition. Furthermore, we considered the potential new therapeutic approaches that can be used to overcome current treatment constraints.

## DEVELOPMENT OF THE HUMAN GI MICROBIOTA

It is believed that in humans, the microbiota begins to co-evolve as a physiologic community consisting of distinct niches in different parts of the body immediately after birth, showing metabolic and antigenic diversity. Many studies have investigated this phenomenon, going so far as to detect microbes even in womb tissues [9,10]. Following delivery, the body is colonized by microorganisms, creating a uniquely structured microbiota based partly on life events, such as the mode of delivery, illness, antibiotics usage, diet, geographical location, and general lifestyle [10,11].

Microbial colonization is influenced by mode of delivery. During the first few days of life, infants that are vaginally delivered are highly colonized with members of the genus *Lactobacillus* (belonging to phylum Firmicutes) compared to infants delivered via cesarean section (C-section) owing to the high abundance of lactobacilli in the vaginal niche [12,13]. Microbial colonization of infants delivered by C-section is reduced and delayed, because they are deprived of contact with maternal vaginal microbiota,, particularly of obligate anaerobic bacteria, such as Bacteroides and Bifidobacterium [14-16]; babies delivered by C-section are more likely to have immune-mediated disorders [17,18]. In terms of the GI tract, 75% of the stool microbiota of vaginally delivered babies are similar to their mothers’ stool microbiota, whereas in babies delivered by C-section this fraction is considerably lower (41%) [19]. Generally, the composition of the microbiota in the early stages of life has low diversity and is dominated by two phyla—Actinobacteria and Proteobacteria [10,20]. Starting from the first few months of life and up to the time of exposure to solid foods, a well-characterized range of stereotypic microbial structures appear in the intestinal feces, where the microbial diversity gradually increases. This suggests that microbial colonization is acquired from sources other than, or in addition to, what is inherited from family members [21]. By the end of the first 3 years of life, the diversity and functional capacities of the microbiota develop towards a distinctive adult-like microbial profile that comprises a temporal pattern unique to each individual.

Despite the relative stability of the gut microbiota in adulthood, it is predisposed to perturbation over time with respect to life events [22]. Therefore, descriptions of the adult microbiota as “stable” are not entirely accurate, owing to the ongoing coexistence of some local species [15,21]. Notably, microbiota in individuals over the age of 65 have shown to shift towards an increased proportion of Bacteroidetes and Firmicutes, especially *Clostridium* cluster IV, compared to younger subjects, where cluster XIVa is more common [23]. However, in another study, the diversity of the microbiota of elderly individuals aged over 100 years was found to be significantly decreased in a cohort of patients, exhibiting profound specific variations, such as an expansion in the abundance of facultative anaerobes (e.g., *Escherichia coli*) and a re-arrangement of the profile of short-chain fatty acid (SCFA) producers in particular butyrate-producing bacteria (e.g., *Faecalibacterium* *prausnitzii*) [24]. Lifestyle interactions, such as community-dwelling and long-standing residential care, are the main factors that affect the diversity and composition of microbiota in elderly populations [25]. Furthermore, the overall metabolic capacity of the microbiota in elderly individuals is altered. For example, the microbiota of elderly individuals tend to have reduced SCFA production and amylolysis, as well as increased proteolytic activity [26]. Since there is a large body of evidence suggesting that SCFAs play a major role in metabolic activities and act as immune mediators, alterations in microbiota may explain the observed increase in inflammatory-ageing that occurs in the GI tract of elderly individuals [27].

## A HEALTHY MICROBIOME

Although indigenous microbiota are now recognized as an important aspect of human health, the phylogenetic and functional composition of a healthy microbiome has yet to be precisely identified [28]. Healthy microbial patterns have not been conclusively documented and functional descriptions of healthy microbiota are less clear than disease-associated microbial patterns. While there is agreement that microorganisms, particularly bacteria, play an important role in health and disease, no clear causal relationships have been established [28]. Perhaps the best description of a healthy microbiome is the ecological stability of microbial patterning on or in the body habitat or body sites. Hence, preserving the resilience of bacterial populations to ecologic stress or perturbation may be an important consideration. The persistent variation found in microbiota between individuals suggests that the idea of defining a healthy microbiome as a specific collection of bacterial species within a community is too simplistic. An alternative concept of a healthy microbiome would describe functional genes and the presence of metabolic pathways [28]; this would better explain the differences in and between healthy and unhealthy people.

In healthy states and under normal circumstances, microbes within the GI tract perform beneficial functions for the human host. The GI environment supports the growth, reproduction, and longevity of the microbial community [29]. In adulthood, when the composition of the GI microbiota is assumed to be diverse and stable, a large variety of microbes, comprising over 1000 different bacterial species coexist. Among these, four dominant bacterial phyla have been identified via molecular-dependent methods: Bacteroidetes, Firmicutes, Protobacteria, and Actinobacteria [30,31].

In addition to bacteria, the GI microbiota include archaea, in particular *Methanobrevibacter smithii*, found in almost 96% of healthy individuals; these produce methane from the hydrogen generated by bacterial catabolism [32]. Furthermore, fungi, such as members of the phyla Ascomycota and Basidiomycota, are also an important portion of the human GI microbiota [33]. *Saccharomyces*, *Candida*, and *Cladosporium* are human-associated fungal genera that include several low-abundance strains. Notably, *M. smithii*, *Saccharomyces*, and *Candida* are frequently found together in individuals that have carbohydrate-rich diets [33]. Even though *Candida* is considered part of the normal flora and can remain non-pathogenic in many individuals, the usage of antibiotics or immunosuppressants can encourage outgrowth and niche-specific invasion by this genus throughout host tissues and mucosa [33].

There are complex interaction networks among the main microbial phyla present in the gut, which reflect the range of dynamic exchanges needed for a physiological microbiota-host balance [34]. Analysis of the human microbiome by the Human Microbiome Project has revealed that, besides the most dominant phyla (Bacteroidetes and Firmicutes), there is a large degree of variation in the relative composition of healthy microbiota, both in phylum-genus distributions and in terms of individual differences, that were initially grouped into different enterotypes [35].

Generally, the mammalian immune system has complicated dynamic bidirectional interactions with the host-associated microbiota. Although recent studies suggest that most immune responses are derived from stimulation of the host genome, almost 10% of immune responses are stimulated by direct interaction with the host microbiome [36,37].

There has been significant debate regarding the temporal stability of the microbiota in healthy individuals, since many factors affect it (e.g., environment [38], travel [39], interactions with other humans or pets [40], diet [41], antibiotics [42], and tobacco use [43]). Also, the rate of change of the composition of microbial populations varies between individuals [44]. As such, it is important to emphasize that there is no general formula for a healthy microbiota that reflects microbial functional redundancy across microbiota-host relationships.

## DYNAMICS OF THE HUMAN MICROBIOME

In 2005, human health was defined by Bircher et al. as “a dynamic state of well-being characterized by a physical, mental, and social potential, which satisfies the demands of life commensurate with age, culture, and personal responsibility” [28,45]. Therefore, the dynamics of the microbiota in response to interactions with other humans, with other non-human creatures, or with the surrounding environment should be taken into consideration when studying the healthy microbiome. Studies investigating the role of the microbiome in human health monitored the dynamics of the changes that occur in microbial communities, demonstrating both plasticity and stability in the human microbiome [21,46]. In these studies, samples (of oral, skin, and GI microbiota) were collected daily from two individuals over the periods of 6 and 15 months. Only a fraction of microbial taxa was persistently present across all (or most) samples from the donor hosts. For example, left and right palm skin samples showed no persistently present species, whereas in the intestinal and oral samples approximately 5% of the detected species were identified as belonging to a core microbiota that was relatively stable. These findings support the notion of the individuality of each human microbiome.

The uniqueness of an individual’s microbiome significantly surpasses that of the host genome, which is roughly 99.5% indistinguishable between people. Since the composition of the microbiome varies considerably, microbiome analysis can be successfully used for forensic purposes [47]. Moreover, besides the high degree of personalization in the microbiome, variations in the rate of change of the microbiome between individuals may be significant and should not be neglected [44].

An important observation is that no significant changes in the compositions of the microbiome have been observed between individuals who used antibiotics and those who did not on the day of sampling (or during the previous week). This suggests that a one-week duration is not adequate to accurately capture the effect of antibiotics use on microbiome composition. Nevertheless, the use of antibiotics is associated with considerable modifications in the composition of the microbiome [37].

## IBD-ASSOCIATED MICROBIAL PATTERNS

The incidence of IBD has been increasing globally over recent years, but there is not sufficient evidence to comprehensively explain its etiology [48]. The most accepted theory of IBD pathogenesis involves interactions between host genetics, immune systems, and environmental factors that drive aberrant inflammatory immunological responses [49]. From a genetic point of view, variations within more than 200 genes have been implicated in IBD development [50]. For example, the *NOD2* and *ATG16L1* genes are thought to contribute to imperfections in the function of the epithelial barrier as well as of microbial recognition and clearance [51], which implicate intestinal microbes as drivers of IBD-associated inflammation.

Studies have found that IBD usually appears following alterations in the gut microbiota (i.e., dysbiosis) [37]. Dysbiosis, described at a functional level, is the failure of the microbiota to provide the host with the full complement of beneficial properties [52]. According to the IBD dysbiosis theory, changes in the composition and localization of GI microbiota are apparent in patients with confirmed IBD compared to healthy individuals [53,54]. In general, reports suggest that the intestinal microbiota of IBD patients have less biodiversity as well as taxonomic and functional shifts, which seem to be hallmarks of IBD [55].

In fact, there is a complex interaction between the intestinal epithelial cells, the host immune system, and the abundance of gut microbiota. Hence, many factors can contribute to the onset of inflammation:

1. Alterations in the balance of commensal and pathogenic microbiota may result in excessive production of pro-inflammatory compounds and lead to exacerbated intestinal inflammation [56]. Many studies based on the analysis of fecal samples of IBD patients have noted a reduction in the frequency of Bacteroidetes and Firmicutes and an increase of Proteobacteria and Actinobacteria [57,58]. In IBD patients, advances based on metagenomic sequencing of microbial RNA have found a decline in bacterial composition and diversity when compared to unaffected individuals [59].
2. Deficiencies in the integrity of the epithelial barrier can lead to an increase in luminal antigen uptake, which ultimately leads to continuous immune activation [56].
3. Research has demonstrated that decreased mucin production, due to depletion of the goblet cells and dysfunction of the epithelial cell tight junction, is also involved in the pathophysiology of IBD [59].
4. One of the most highlighted hallmarks of IBD, particularly in CD patients, is the significant decrease of *F. prausnitzii*, which is a member of Firmicutes phylum and one of the most abundant bacterial species in the GI tract (especially in the colon) of healthy individuals [53,54,59-62].

These observations support the major role of microbial dysbiosis in the induction of IBD [63,64]. The role of the microbiome in IBD pathogenesis and therapy suggests that antibiotic treatments can lead to improvements in IBD patients [65,66]. For example, treating UC patients with antibiotics diminishes mucosal inflammation [59]. Other strategies to re-establish the microbiota in IBD patients are available. For example, improvement in inflammation and mucosal recovery was observed as a result of fecal transplantation in CD patients, although this was followed by disease reactivation [67]. However, there is still uncertainty whether dysbiosis has a causative or an associative relationship with IBD, likely because the majority of investigations into the intestinal microbiota of IBD populations have been conducted following the onset of the disease [68].

Current literature investigating IBD and accompanying alterations in microbiota often fails to highlight the difference between changes to the mucosal and fecal microbiota, regardless of confounding factors, such as chronicity of disease, therapeutic approaches, and surgical intervention [69]. Therefore, it is still difficult to decipher whether taxonomic modifications reveal disease-associated changes or are a response to a changed intestinal environment. The first study to investigate dysbiosis in IBD, while controlling for previously identified confounding factors, assessed changes to the mucosal ­microbiota in 13 and 12 children with newly diagnosed CD and UC, respectively [70]. All patients were assessed at the time of the initial presentation of active colitis. During the 3 months prior to diagnosis, no antibiotics or steroids were given and no immunosuppression drugs were prescribed. The results showed a significant reduction in microbial diversity in CD patients compared to UC patients and the control population. Surprisingly, an increase in *F. prausnitzii* abundance was observed in CD patients, which was markedly dissimilar to the findings of several other studies [57,71-75].

Hypothetically, serial, and comparative follow-ups of such patient cohorts enable a distinct chance to examine clear and accurate profiles of IBD associations with microbiota alterations. Outcomes of the previous studies regarding alterations in the microbiota that accompany IBD (in either CD or UC) are summarized in Table 1.

**TABLE 1:**
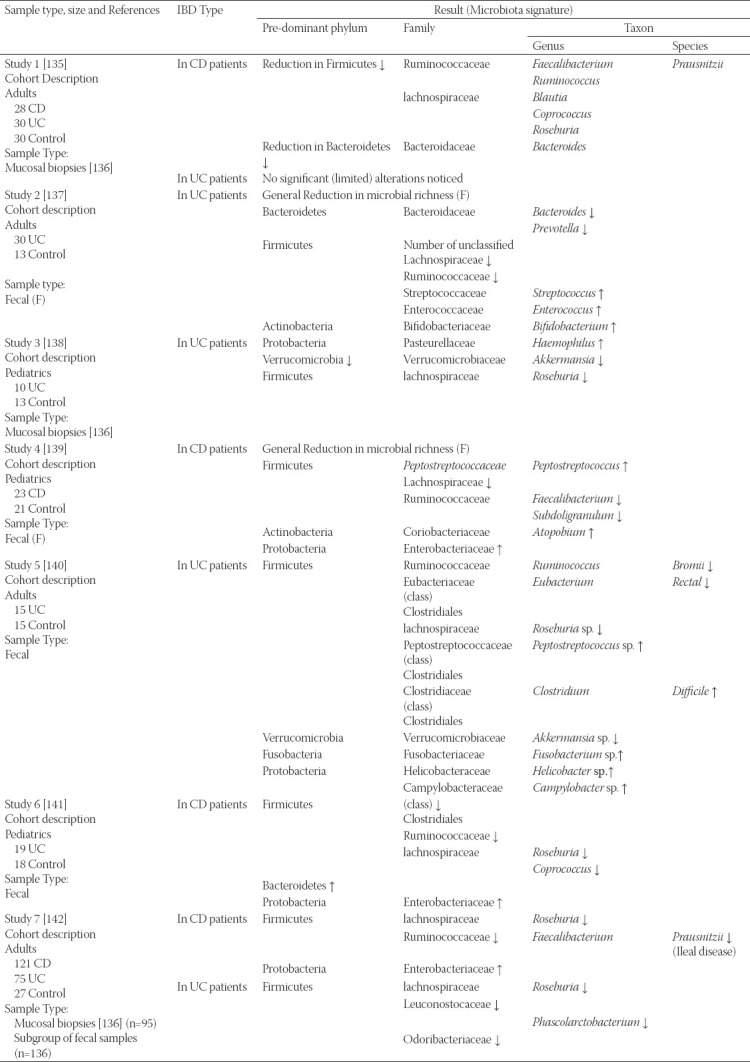
Findings of several studies concerned with gut microbiota dysbiosis in IBD. F = changes within fecal samples, T = changes within tissue/mucosal (biopsy samples), reproduced from Mcllroy et al. [134]

## IMMUNE DYSFUNCTION IN IBD

As mentioned above, IBDs are believed to result from an abnormal immune response to GI tract microbiota in genetically susceptible individuals, although with unclear interactions between the causative factors [63]. Indirect evidence for the involvement of microbiota in the pathogenesis of IBDs includes studies that have shown evidence of mucosal T cells react against GI tract microbiota [76], and mucosal secretion of immunoglobulin G antibodies in patients with confirmed IBD [77]. UC and CD seem to be histologically distinct, since inflammation in UC is superficial and limited to the colon, while in CD the inflammation is generally transmural, multi-focal and can contain granulomas. Immunologically, UC and CD are also separate. In general, “helper” T cells are divided into Th1 and Th2 subsets, depending on the cytokines types that they produce. This observation was reported in the 1980s in mice, so may not be equitable with human T cells. However, in general, CD can be regarded as a Th1-type inflammatory process, with increased expression of IFN-γ and IL-2, in addition to the Th1-inducing cytokine, IL-12. The UC cytokine profile is different. There is increased expression of IL-5 and IL-13, and cytokines are more commonly associated with a Th2 response; however, the archetypal Th2 cytokine IL-4 is not upregulated, and increased levels of IFN-γ are seen [78]. Latterly, non-Th1/Th2 pathways have been characterized to be involved in IBD pathogenesis. Another interleukin, IL-23, is an inducer of a different subset of pro-inflammatory T cells, known as Th17 cells, owing to their high level of IL-17 production [79]. High expression of IL-17 has been reported in active CD and UC and may be of potential as a future therapeutic target [79].

Of particular note, it has also been found that overproduction of the tumor necrosis factor (TNF) by CD14+ macrophages, fibroblasts, and T cells is associated with IBD pathogenesis. TNF enhances several pro-inflammatory properties in chronic intestinal inflammation, including epithelial cell damage [80]. Importantly, a number of pro-inflammatory effects are mediated by membrane-bound TNF, instead of soluble TNF, which indicates the therapeutic potential of targeting the pathway of the membrane-bound TNF and its receptor, TNF-R2 [81]. Accordingly, an improvement of the gut inflammation in mice with IBD was observed after treatment with anti-TNF. Clinically, studies have revealed the effectiveness of suppressing the TNF effect by neutralizing its antibodies in CD and UC [82]. Hence, a number of antibodies to TNF that target soluble TNF plus membrane-bound TNF, such as infliximab (IFX) and adalimumab (ADA) are at present in routine clinical use for IBD treatment [83]. IFX is a therapeutic monoclonal antibody against TNF-α used for patients with moderate-to-severe Crohn’s disease. However, many IBD patients show no response to anti-TNF treatment or it may lose clinical response effectiveness over time, which prompts the development of novel therapeutic approaches [84].

## IS THE GUT MICROBIAL DYSBIOSIS THAT ACCOMPANIES IBD A CAUSE OR A CONSEQUENCE?

It has been difficult to find clear evidence whether dysbiosis observed with IBD is a cause or an outcome of the disease. This point must be addressed since it has direct implications on therapeutic drug development and diagnostic and prognostic investigations, as well as on strategies used to predict individuals’ response to therapies [85].

On the one hand, based on observation, dysbiosis is considered a cause of IBD, with T-cells as mediators of chronic experimental inflammation [86,87]. For instance, mice were observed to develop inflammation after being given transferred stools of mice with colitis [88]. Additionally, CD patients that underwent a fecal transplantation procedure showed a reduction in inflammation and an enhancement of the role of certain probiotic combinations [89]. Although dysbiosis may be present at initial stages before disease progression and treatment administration, it is thought that it may be caused by earlier usage of antibiotics [73,90].

On the other hand, some studies have suggested that dysbiosis is a consequence of combined factors, including inflammation, antibiotics usage, and dietary intake, which affect the homeostasis of microbial communities [91]. A number of studies have revealed that ileal inflammation has a direct impact on bacterial composition and also alters gene expression [92-95]. A study conducted on mice demonstrated that alterations in gene expression of enteric species were delivered by inflammation, while disruptions targeting *E. coli* genes, which are typically provoked by inflammation; limit the severity of inflammation [92,96]. Additionally, the increased association of some intestinal microbes (e.g., *E. coli*) with inflammation in IBD patients can be explained by alterations in epithelial defenses, as well as by mucosal thickness and viscosity caused through the inflammation [97]. Highly damaged intestinal regions with ulcerations potentially facilitate the accession of invasive, oxygen-tolerant microbes [98].

## PRESENT TREATMENTS FOR IBD

The utilization of therapeutic options for IBD has long been restricted by an imprecise understanding of the disease etiology. The hypothesis that the IBD is caused by complex genetic and environmental interactions leading to excessive production of pro-inflammatory cytokines and terminates with inflammation has advanced certain therapeutic approaches [99]. Several drugs that are widely used to treat IBD target the pathological over-active immune responses of individuals rather than other possible underlying factors [100]. This explains the use of immunosuppressive therapies [99] such as steroids, although an argument exists that these treatments do not specifically target the aberrant immune responses or GI microbiota [101] as effectively as immunomodulators (e.g., azathioprine and methotrexate) and biologics [63,102]. Biologics used to treat IBD either target anti-adherent pro-inflammatory cytokines, such as TNF-α and or interleukins, such as IL-12/IL23, to block inflammation or prevent recruitment of immune cells into the intestinal tissues, which can be achieved with leukocyte trafficking inhibitors. A central goal of IBD therapies is inducing and maintaining mucosal healing [103,104]. Thus, assessment of the therapeutic novelty and efficacy of any new therapy is based on its ability to stimulate mucosal healing. However, for these assessments to be valid it is important to precisely define mucosal healing and how mucosal healing affects long-term disease, both of which have been substantially debated [99].

## WHAT IS MUCOSAL HEALING?

**FIGURE 2:**
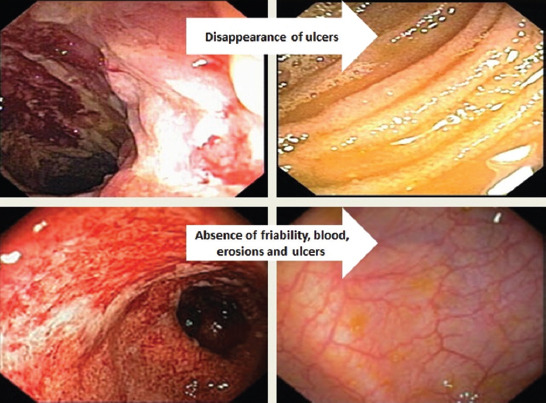
Sequential mucosal healing process of the terminal ileum and the colon in patients with CD and UC.

While monitoring disease activity to assess the efficacy of IBD treatments in clinical practice and for endpoints in clinical trials is indispensable to therapeutic development, relying solely on clinical symptoms provides inadequate descriptions of IBD. Mucosal healing is measured by assessing the colonic and intestinal mucosa for active inflammation using endoscopy [99], which is characterized by the absence of ulceration in CD [105]. There has been some debate regarding the definition of mucosal healing in UC, but the most agreed upon definition is based on the Mayo endoscopic scoring system and refers to the absence of friability, erosions, ulcerations, and spontaneous bleeding of the colonic mucosa (Figure 2) [104,106].

Currently, C-reaction protein (CRP) [107] and fecal calprotectin [108] are used as additional measures for examining disease activity [107]. Although they are not targeted by treatment, they are valuable and informative indicators that can be used to monitor IBD patients. Histopathological analysis is also used as a measure of intestinal inflammation [109-111]. Interestingly, some recent findings suggest that the efficacy of histologic healing in predicting long-term outcomes in UC patients surpasses endoscopic documentation of mucosal healing [99].

## INFLUENCE OF TNF-α ANTAGONIST THERAPY ON GI MICROBIOTA

TNF-α is a transmembrane, pro-inflammatory cytokine that has been implicated in the immunopathogenesis of CD and many other inflammatory and autoimmune diseases. Anti-TNF inhibitors are used to treat moderate-to-severe CD patients, as the reduction of TNF-α levels by anti-TNF agents leads to a reduction in the chronic pathologic inflammatory responses that characterizes the disease [112,113]. TNF-α is expressed on the surface of macrophages, T-lymphocytes, polymorphonucleated, intestinal epithelial cells, endothelial cells, and natural killer cells [114].

The development of anti-TNF-α therapies marked the beginning of pathway-based therapies, or what are commonly termed antibody-based therapies, and initiated a new era of targeted treatment. Since 1998, the anti-TNF-α antibody IFX has been approved by the Food and Drug Administration (FDA) in the United States for treatment of UC. The FDA approved the efficacy of its ability in treating UC [115], since these are promising therapeutic approaches which target pro-inflammatory cytokines (IL12/IL23) [84,99,116]. Subsequently, many other agents of this class have emerged onto the market and have shown a positive impact on intestinal inflammation, including the induction and maintenance of mucosal healing [117]. The most frequently used anti-TNF agents in clinical practice are IFX and ADA [118,119].

Notwithstanding the considerable success achieved by antibody-based treatments, there remains a largely unmet clinical need for novel therapeutic approaches for the subgroups of patients that fail to respond to IFX or ADA (primary non-response) or show loss of response over time (secondary-loss of response) [99]. For example, about 30% of CD patients show no response to the anti-TNF antibody, whereas 50% experience a steady loss of response to anti-TNF therapy after primary clinical response [113].

According to the microbiota dysbiosis theory of IBD pathogenesis, effective treatments for IBD should somehow affect the composition of gut microbiota. It is possible that the dysbiosis that has been reported as a hallmark of IBD can be restored to a normal state in patients that respond to anti-TNF therapies. However, there is insufficient evidence of a positive relationship between anti-TNF drugs and rebalancing of the GI microbiota composition or the impact of anti-TNF drugs on microbiota in long-term disease outcomes.

**FIGURE 3:**
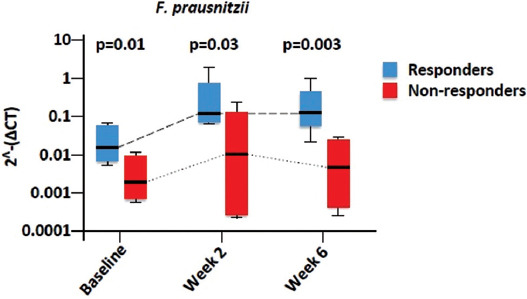
*Faecalibacterium prausnitzii* in responders to anti-TNF compared to non-responders, reproduced from Yoshihara et al. [113].

In a study investigating the effect of using anti-TNF therapies on the fecal microbiota composition of UC patients, fecal samples at baseline were obtained from four patients who responded to treatment and from three patients that were primary non-responders [120]. Samples were also collected from eight responders and seven non-responders at week 2, as well as from eight responders and five non-responders at week 6. The study found that responders had lower average dysbiosis scores than non-responders. Also, a higher abundance of *F. prausnitzii* was observed in responders at every time point (Figure 3). Recolonization of *F. prausnitzii* is known to be associated with maintaining remission after CD relapse [121].

A study by Li et al. investigated Chinese pediatric patients with CD who were treated with IFX, assessing the dynamic changes in microbiota during treatment and the influence of the anti-TNF agent on the composition of microbiota. Their results suggested that IFX altered the structure of the gut microbiome and its metabolic function. Furthermore, they found that CD-correlated GI microbial dysbiosis was characterized by a significant increase in the number of SCFA-producing bacterial taxa, such as *Anaerostipes*, *Blautia*, *Coprococcus*, *Faecalibacterium*, *Lachnospira*, *Odoribacter*, *Roseburia*, *Ruminococcus*, and *Sutterella* [122].

Although immunosuppressive drugs have been proven to be effective for the treatment of IBD, significant limitations in these therapies have been found, such as being successful only in subsets of patients and with risks of adverse effects [123]. There is, thus, a need for non-immunosuppressive treatments that selectively focus on the distribution of gut microbiota related to IBD [100]. Studying how these therapies may perturb and or reconstitute the gut microbiota is essential to determine what aspects of the structural changes to the gut microbiome in active colitis and treatment-induced remission can be targeted by therapeutics.

Broadly, there is evidence of intestinal dysbiosis in IBD and evidence for the symbiotic relationship between intestinal microbiota and the intestinal immune system. Current therapies are expected to influence the diversity of gut microbiota in treated patients and there is potential for microbiome-modulating therapeutic approaches in preventing relapsing colitis.

## POTENTIAL THERAPIES FOR IBD BY MODULATING MICROBIOME COMPOSITION AND FUNCTION

The main goal of current IBD therapeutic approaches is the induction and maintenance of remission. Continued remission of the disease can be achieved through traditional treatments, such as corticosteroids or biologics, combined with some physiologic, and less toxic treatments that can specifically modulate microbiota composition. One approach for treating IBD is to initially target inflammation by modulating the microbiota. This alternative therapeutic approach, which is called microbiome-modulating therapy [124], can enable the correction of dysbiosis, restitution of normal microbial function, normalization of the immune system responses, and repair of epithelial barrier deficiencies. Therefore, there is an increasing need for developing novel therapeutic approaches that can fully cure or even prevent IBD.

Many other promising approaches have been suggested for treating IBD, such as probiotics, prebiotics, fecal microbiota transplantation, synthetic combinations of specific bacteria, and personalized therapies based on individual microbiome profiles; the latter customizes a patient’s diet and applies highly selective antibiotics for major aggressive bacterial species. Other novel but hypothetical approaches include stimulation of the protective host pathways via synthetic microbial metabolites or using recombinant bacterial species, utilizing bacteriophages to target aggressive microbes, and disabling bacterial attachment and blocking bacterial receptors, improving the anaerobic environment for growing anaerobic bacteria [125,126]. This latter approach is based on the finding that reduction in *F. prausnitzii* has been proven to decrease the production of butyrate in IBD patients, which is considered to be the main source of oxygen for epithelial cells. This leads to the production of energy from the fermentation of glucose to lactose, which increases oxygen levels to up to 3-10% in the GI tract environment. Subsequently, the balance of the local bacterial community is affected as the strict anaerobic environment gradually decreases and facultative anaerobes, such as *E. coli*, proliferate [49].

Collectively, researchers and physician hope that investigating and manipulating the intestinal microbiota can help to discern the cause or causes behind the relatively recent increase in incidence of IBD.

## CAN MICROBIOME COMPOSITION BE A PREDICTOR OF CLINICAL RESPONSE TO ANTI-TNF?

Within a population, the microbiome exhibits a larger degree of variance with disease than host genetics. Several studies of different diseases have also highlighted the predictive capacity of the microbiome as a biomarker. The total structure and diversity of the microbiota, or even the existence or loss of specific taxa, have been demonstrated to be biomarkers of illnesses or can be used to speculate about potential treatments in several diseases [127].

**FIGURE 4:**
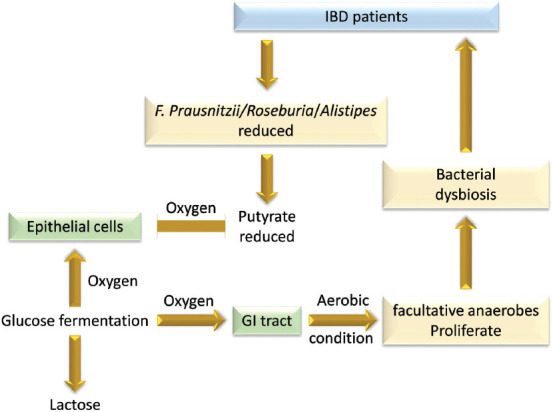
Consequences of reduction of butyrate-producing bacteria on the balance of local bacterial community in the GI tract of IBD patients.

For example, the abundance of *F. prausnitzii* in the ileum of CD patients has been linked to the risk of post-operative recurrence [57]. In UC patients, the risk of pouchitis post-colectomy can be predicted by bacterial configuration [128]. In addition, an association between general dysbiosis of the microbiota and relapse following IFX treatment has been postulated [129]. In the present study, dysbiosis was observed in CD patients and was typified by low *F. prausnitzii* (*p* = 0.003), and a reduction in Firmicutes was identified in relapsers. Furthermore, a low rate of *F. prausnitzii* (*p* = 0.014) and a low rate of bacteroides (*p* = 0.030) were also predictive of relapse. Similarly, the absence of *Roseburia* and *F. prausnitzii*, both butyrate-producing bacteria and the abundance of *E. coli* were found to be linked with an ileal CD phenotype [53]. Another butyrate-producing bacterium, *Alistipes*, was found to be considerably depleted in IBD patients [130]. These confirm the importance of acetate-to-butyrate conversion bacteria in maintaining GI homeostasis, (Figure 4) [53,130,131].

## CONCLUSION

Owing to the importance of the symbiotic relationship between the intestinal microbiota and the host, it is understandable that shifts in gut microbiota have been implicated in various diseases, ranging from GI conditions, such as IBD, to neurodevelopmental diseases, such as autism [132,133]. Dysbiosis appears to exacerbate the progression of IBD, either as a cause or as a consequence. Restoring the microbiota composition in patients depends on the type and stage of the disease. Reconstitution of the microbiome, taking the host genetic factors into consideration, can theoretically be achieved through combining immunosuppressants (e.g., anti-TNF drugs) and microbiota-modulator therapies (e.g., antimicrobials, diet, prebiotics or probiotics, and FMT). These dual therapeutic approaches may help in restoring the ideal environment needed to re-induce an effective symbiotic relationship between the host and beneficial microbes. Thus, the success of therapeutic strategies relies on understanding how microbiota interact with the host.

## References

[1] Ng SC, Shi HY, Hamidi N, Underwood FE, Tang W, Benchimol EI (2017). Worldwide incidence and prevalence of inflammatory bowel disease in the 21^st^ century:A systematic review of population-based studies. Lancet.

[2] Turnbaugh PJ, Ley RE, Hamady M, Fraser-Liggett CM, Knight R, Gordon JI (2007). The human microbiome project. Nature.

[3] Bouma G, Strober W (2003). The immunological and genetic basis of inflammatory bowel disease. Nat Rev Immunol.

[4] Kovarik JJ, Tillinger W, Hofer J, Holzl MA, Heinzl H, Saemann MD (2011). Impaired anti-inflammatory efficacy of n-butyrate in patients with IBD. Eur J Clin Invest.

[5] Olivera P, Danese S, Jay N, Natoli G, Peyrin-Biroulet L (2019). Big data in IBD:A look into the future. Nat Rev Gastroenterol Hepatol.

[6] Ni J, Wu GD, Albenberg L, Tomov VT (2017). Gut microbiota and IBD:Causation or correlation?. Nat Rev Gastroenterol Hepatol.

[7] Jones-Hall YL, Nakatsu CH (2016). The intersection of TNF, IBD and the microbiome. Gut Microbes.

[8] Cozzi S, Escarpa AS, Parra DL, Jamal DN, Mitjana JM, Piulats JM (2019). Association between inflammatory bowel disease and uveal melanoma:Case report of two young adults and a literature review. Rep Pract Oncol Radiother.

[9] Aagaard K, Ma J, Antony KM, Ganu R, Petrosino J, Versalovic J (2014). The placenta harbors a unique microbiome. Sci Transl Med.

[10] Rodríguez JM, Murphy K, Stanton C, Ross RP, Kober OI, Juge N (2015). The composition of the gut microbiota throughout life, with an emphasis on early life. Microb Ecol Health Dis.

[11] Koenig JE, Spor A, Scalfone N, Fricker AD, Stombaugh J, Knight R (2011). Succession of microbial consortia in the developing infant gut microbiome. Proc Natl Acad Sci.

[12] Avershina E, Storrø O, Øien T, Johnsen R, Pope P, Rudi K (2014). Major faecal microbiota shifts in composition and diversity with age in a geographically restricted cohort of mothers and their children. FEMS Microbiol Ecol.

[13] Aagaard K, Riehle K, Ma J, Segata N, Mistretta TA, Coarfa C (2012). A metagenomic approach to characterization of the vaginal microbiome signature in pregnancy. PLoS One.

[14] Jakobsson HE, Abrahamsson TR, Jenmalm MC, Harris K, Quince C, Jernberg C (2014). Decreased gut microbiota diversity, delayedBacteroidetes colonisation and reduced Th1 responses in infants delivered by caesarean section. Gut.

[15] Costello EK, Stagaman K, Dethlefsen L, Bohannan BJ, Relman DA (2012). The Application of ecological theory toward an understanding of the human microbiome. Science.

[16] Salminen S, Gibson G, McCartney A, Isolauri E (2004). Influence of mode of delivery on gut microbiota composition in seven year old children. Gut.

[17] Kuitunen M, Kukkonen K, Juntunen-Backman K, Korpela R, Poussa T, Tuure T (2009). Probiotics prevent IgE-associated allergy until age 5 years in cesarean-delivered children but not in the total cohort. J Allergy Clin Immunol.

[18] van Nimwegen FA, Penders J, Stobberingh EE, Postma DS, Koppelman GH, Kerkhof M (2011). Mode and place of delivery, gastrointestinal microbiota, and their influence on asthma and atopy. J Allergy Clin Immunol.

[19] Bäckhed F, Roswall J, Peng Y, Feng Q, Jia H, Kovatcheva-Datchary P (2015). Dynamics and stabilization of the human gut microbiome during the first year of life. Cell Host Microbe.

[20] Bäckhed F (2011). Programming of host metabolism by the gut microbiota. Ann Nutr Metab.

[21] Caporaso JG, Lauber CL, Costello EK, Berg-Lyons D, Gonzalez A, Stombaugh J (2011). Moving pictures of the human microbiome. Genome Biol.

[22] Dethlefsen L, Relman DA (2011). Incomplete recovery and individualized responses of the human distal gut microbiota to repeated antibiotic perturbation. Proc Natl Acad Sci.

[23] Claesson MJ, Cusack S, O'Sullivan O, Greene-Diniz R, de Weerd H, Flannery E (2011). Composition, variability, and temporal stability of the intestinal microbiota of the elderly. Proc Natl Acad Sci.

[24] Biagi E, Nylund L, Candela M, Ostan R, Bucci L, Pini E (2010). Through ageing, and beyond:Gut microbiota and inflammatory status in seniors and centenarians. PLoS One.

[25] Claesson MJ, Jeffery IB, Conde S, Power SE, O'connor EM, Cusack S (2012). Gut microbiota composition correlates with diet and health in the elderly. Nature.

[26] Woodmansey EJ, McMurdo ME, Macfarlane GT, Macfarlane S (2004). Comparison of compositions and metabolic activities of fecal microbiotas in young adults and in antibiotic-treated and non-antibiotic-treated elderly subjects. Appl Environ Microbiol.

[27] Biagi E, Candela M, Turroni S, Garagnani P, Franceschi C, Brigidi P (2013). Ageing and gut microbes:Perspectives for health maintenance and longevity. Pharmacol Res.

[28] Bäckhed F, Fraser CM, Ringel Y, Sanders ME, Sartor RB, Sherman PM (2012). Defining a healthy human gut microbiome:Current concepts, future directions, and clinical applications. Cell Host Microbe.

[29] Browne HP, Forster SC, Anonye BO, Kumar N, Neville BA, Stares MD (2016). Culturing of “unculturable”human microbiota reveals novel taxa and extensive sporulation. Nature.

[30] Qin J, Li R, Raes J, Arumugam M, Burgdorf KS, Manichanh C (2010). A human gut microbial gene catalogue established by metagenomic sequencing. Nature.

[31] Guinane CM, Tadrous A, Fouhy F, Ryan CA, Dempsey EM, Murphy B (2013). Microbial composition of human appendices from patients following appendectomy. MBio.

[32] Lurie-Weinberger MN, Gophna U (2015). Archaea in and on the human body:Health implications and future directions. PLoS Pathog.

[33] Hoffmann C, Dollive S, Grunberg S, Chen J, Li H, Wu GD (2013). Archaea and fungi of the human gut microbiome:Correlations with diet and bacterial residents. PLoS One.

[34] Lagkouvardos I, Overmann J, Clavel T (2017). Cultured microbes represent a substantial fraction of the human and mouse gut microbiota. Gut Microbes.

[35] The Human Microbiome Project Consortium (2012). Structure, function and diversity of the healthy human microbiome. Nature.

[36] Schirmer M, Smeekens SP, Vlamakis H, Jaeger M, Oosting M, Franzosa EA (2016). Linking the human gut microbiome to inflammatory cytokine production capacity. Cell.

[37] Gilbert JA, Blaser MJ, Caporaso JG, Jansson JK, Lynch SV, Knight R (2018). Current understanding of the human microbiome. Nat Med.

[38] Ursell LK, Van Treuren W, Metcalf JL, Pirrung M, Gewirtz A, Knight R (2013). Replenishing our defensive microbes. Bioessays.

[39] Rajilić-Stojanović M, Heilig HG, Tims S, Zoetendal EG, de Vos WM (2012). Long-term monitoring of the human intestinal microbiota composition. Environ Microbiol.

[40] Song SJ, Lauber C, Costello EK, Lozupone CA, Humphrey G, Berg-Lyons D (2013). Cohabiting family members share microbiota with one another and with their dogs. Elife.

[41] David LA, Maurice CF, Carmody RN, Gootenberg DB, Button JE, Wolfe BE (2014). Diet rapidly and reproducibly alters the human gut microbiome. Nature.

[42] Dethlefsen L, Huse S, Sogin ML, Relman DA (2008). The pervasive effects of an antibiotic on the human gut microbiota, as revealed by deep 16S rRNA sequencing. PLoS Biol.

[43] Biedermann L, Zeitz J, Mwinyi J, Sutter-Minder E, Rehman A, Ott SJ (2013). Smoking cessation induces profound changes in the composition of the intestinal microbiota in humans. PLoS One.

[44] Flores GE, Caporaso JG, Henley JB, Rideout JR, Domogala D, Chase J (2014). Temporal variability is a personalized feature of the human microbiome. Genome Biol.

[45] Bircher J (2005). Towards a dynamic definition of health and disease. Med Health Care Philos.

[46] David LA, Materna AC, Friedman J, Campos-Baptista MI, Blackburn MC, Perrotta A (2014). Host lifestyle affects human microbiota on daily timescales. Genome Biol.

[47] Fierer N, Lauber CL, Zhou N, McDonald D, Costello EK, Knight R (2010). Forensic identification using skin bacterial communities. Proc Natl Acad Sci.

[48] Sharara AI, Al Awadhi S, Alharbi O, Al Dhahab H, Mounir M, Salese L (2018). Epidemiology, disease burden, and treatment challenges of ulcerative colitis in Africa and the Middle East. Exp Rev Gastroenterol Hepatol.

[49] Celiberto LS, Graef FA, Healey GR, Bosman ES, Jacobson K, Sly LM (2018). Inflammatory bowel disease and immunonutrition:Novel therapeutic approaches through modulation of diet and the gut microbiome. Immunology.

[50] Brant SR, Okou DT, Simpson CL, Cutler DJ, Haritunians T, Bradfield JP (2017). Genome-wide association study identifies African-specific susceptibility loci in African Americans with inflammatory bowel disease. Gastroenterology.

[51] Knights D, Lassen KG, Xavier RJ (2013). Advances in inflammatory bowel disease pathogenesis:Linking host genetics and the microbiome. Gut.

[52] Thursby E, Juge N (2017). Introduction to the human gut microbiota. Biochem J.

[53] Willing BP, Dicksved J, Halfvarson J, Andersson AF, Lucio M, Zheng Z (2010). A pyrosequencing study in twins shows that gastrointestinal microbial profiles vary with inflammatory bowel disease phenotypes. Gastroenterology.

[54] Carlsson AH, Yakymenko O, Olivier I, Håkansson F, Postma E, Keita ÅV (2013). *Faecalibacterium prausnitzii* supernatant improves intestinal barrier function in mice DSS colitis. Scand J Gastroenterol.

[55] Wright EK, Kamm MA, Teo SM, Inouye M, Wagner J, Kirkwood CD (2015). Recent advances in characterizing the gastrointestinal microbiome in Crohn's disease:A systematic review. Inflamm Bowel Dis.

[56] Sartor RB (2006). Mechanisms of disease:Pathogenesis of Crohn's disease and ulcerative colitis. Nat Clin Pract Gastroenterol Hepatol.

[57] Sokol H, Pigneur B, Watterlot L, Lakhdari O, Bermudez-Humaran LG, Gratadoux JJ (2008). *Faecalibacterium prausnitzii* is an anti-inflammatory commensal bacterium identified by gut microbiota analysis of Crohn disease patients. Proc Natl Acad Sci USA.

[58] Frank DN, Amand AL, Feldman RA, Boedeker EC, Harpaz N, Pace NR (2007). Molecular-phylogenetic characterization of microbial community imbalances in human inflammatory bowel diseases. Proc Natl Acad Sci.

[59] Wallace KL, Zheng LB, Kanazawa Y, Shih DQ (2014). Immunopathology of inflammatory bowel disease. World J Gastroenterol.

[60] Fujimoto J, Kadara H, Garcia MM, Kabbout M, Behrens C, Liu DD (2012). G-protein coupled receptor family C, group 5, member A (GPRC5A) expression is decreased in the adjacent field and normal bronchial epithelia of patients with chronic obstructive pulmonary disease and non-small-cell lung cancer. J Thorac Oncol.

[61] Rossi O, Khan MT, Schwarzer M, Hudcovic T, Srutkova D, Duncan SH (2015). *Faecalibacterium prausnitzii* strain HTF-F and its extracellular polymeric matrix attenuate clinical parameters in DSS-induced colitis. PLoS One.

[62] Walker AW, Ince J, Duncan SH, Webster LM, Holtrop G, Ze X (2011). Dominant and diet-responsive groups of bacteria within the human colonic microbiota. ISME J.

[63] Strober W, Fuss I, Mannon P (2007). The fundamental basis of inflammatory bowel disease. J Clin Investig.

[64] Eichele DD, Kharbanda KK (2017). Dextran sodium sulfate colitis murine model:An indispensable tool for advancing our understanding of inflammatory bowel diseases pathogenesis. World J Gastroenterol.

[65] De Fazio L, Cavazza E, Spisni E, Strillacci A, Centanni M, Candela M (2014). Longitudinal analysis of inflammation and microbiota dynamics in a model of mild chronic dextran sulfate sodium-induced colitis in mice. World J Gastroenterol.

[66] Gkouskou K, Deligianni C, Tsatsanis C, Eliopoulos AG (2014). The gut microbiota in mouse models of inflammatory bowel disease. Front Cell Infect Microbiol.

[67] D'haens GR, Geboes K, Peeters M, Baert F, Penninckx F, Rutgeerts P (1998). Early lesions of recurrent Crohn's disease caused by infusion of intestinal contents in excluded ileum. Gastroenterology.

[68] Dicksved J, Schreiber O, Willing B, Petersson J, Rang S, Phillipson M (2012). *Lactobacillus reuteri* maintains a functional mucosal barrier during DSS treatment despite mucus layer dysfunction. PLoS One.

[69] McIlroy J, Ianiro G, Mukhopadhya I, Hansen R, Hold G (2018). The gut microbiome in inflammatory bowel disease-avenues for microbial management. Aliment Pharmacol Ther.

[70] Hansen R, Berry SH, Mukhopadhya I, Thomson JM, Saunders KA, Nicholl CE (2013). The microaerophilic microbiota of de-novo paediatric inflammatory bowel disease:The BISCUIT study. PLoS One.

[71] Hansen R, Russell RK, Reiff C, Louis P, McIntosh F, Berry SH (2012). Microbiota of de-novo pediatric IBD:Increased *Faecalibacterium prausnitzii* and reduced bacterial diversity in Crohn's but not in ulcerative colitis. Am J Gastroenterol.

[72] Lopez-Siles M, Khan TM, Duncan SH, Harmsen HJ, Garcia-Gil LJ, Flint HJ (2012). Cultured representatives of two major phylogroups of human colonic *Faecalibacterium prausnitzii* can utilize pectin, uronic acids, and host-derived substrates for growth. Appl Environ Microbiol.

[73] Gevers D, Kugathasan S, Denson LA, Vázquez-Baeza Y, Van Treuren W, Ren B (2014). The treatment-naive microbiome in new-onset Crohn's disease. Cell Host Microbe.

[74] Schwiertz A, Jacobi M, Frick JS, Richter M, Rusch K, Köhler H (2010). Microbiota in pediatric inflammatory bowel disease. J Pediatr.

[75] Fujimoto T, Imaeda H, Takahashi K, Kasumi E, Bamba S, Fujiyama Y (2013). Decreased abundance of *Faecalibacterium prausnitzii* in the gut microbiota of C Rohn's disease. J Gastroenterol Hepatol.

[76] Pirzer U, Schönhaar A, Fleischer B, Hermann E, Büschenfelde KH (1991). Reactivity of infiltrating T lymphocytes with microbial antigens in Crohn's disease. Lancet (London, England).

[77] Macpherson A, Khoo UY, Forgacs I, Philpott-Howard J, Bjarnason I (1996). Mucosal antibodies in inflammatory bowel disease are directed against intestinal bacteria. Gut.

[78] Brown SJ, Mayer L (2007). The immune response in inflammatory bowel disease. Am J Gastroenterol.

[79] Harrington LE, Hatton RD, Mangan PR, Turner H, Murphy TL, Murphy KM (2005). Interleukin 17-producing CD4+effector T cells develop via a lineage distinct from the T helper Type 1 and 2 lineages. Nat Immunol.

[80] Günther C, Martini E, Wittkopf N, Amann K, Weigmann B, Neumann H (2011). Caspase-8 regulates TNF-α-induced epithelial necroptosis and terminal ileitis. Nature.

[81] Perrier C, De Hertogh G, Cremer J, Vermeire S, Rutgeerts P, Van Assche G (2013). Neutralization of membrane TNF, but not soluble TNF, is crucial for the treatment of experimental colitis. Inflamm Bowel Dis.

[82] Neurath MF (2019). Targeting immune cell circuits and trafficking in inflammatory bowel disease. Nat Immunol.

[83] Ordás I, Feagan BG, Sandborn WJ (2011). Early use of immunosuppressives or TNF antagonists for the treatment of Crohn's disease:Time for a change. Gut.

[84] Hanauer SB, Feagan BG, Lichtenstein GR, Mayer LF, Schreiber S, Colombel JF (2002). Maintenance infliximab for Crohn's disease:The ACCENT I randomised trial. Lancet.

[85] Sartor RB, Wu GD (2017). Roles for intestinal bacteria, viruses, and fungi in pathogenesis of inflammatory bowel diseases and therapeutic approaches. Gastroenterology.

[86] Buttó LF, Haller D (2016). Dysbiosis in intestinal inflammation:Cause or consequence. Int J Med Microbiol.

[87] Sartor RB (2010). Genetics and environmental interactions shape the intestinal microbiome to promote inflammatory bowel disease versus mucosal homeostasis. Gastroenterology.

[88] Elinav E, Strowig T, Kau AL, Henao-Mejia J, Thaiss CA, Booth CJ (2011). NLRP6 inflammasome regulates colonic microbial ecology and risk for colitis. Cell.

[89] Gionchetti P, Rizzello F, Venturi A, Brigidi P, Matteuzzi D, Bazzocchi G (2000). Oral bacteriotherapy as maintenance treatment in patients with chronic pouchitis:A double-blind, placebo-controlled trial. Gastroenterology.

[90] Huttenhower C, Knight R, Brown CT, Caporaso JG, Clemente JC, Gevers D (2014). Advancing the microbiome research community. Cell.

[91] Lewis JD, Chen EZ, Baldassano RN, Otley AR, Griffiths AM, Lee D (2015). Inflammation, antibiotics, and diet as environmental stressors of the gut microbiome in pediatric Crohn's disease. Cell Host Microbe.

[92] Patwa LG, Fan TJ, Tchaptchet S, Liu Y, Lussier YA, Sartor RB (2011). Chronic intestinal inflammation induces stress-response genes in commensal *Escherichia coli*. Gastroenterology.

[93] Eun CS, Mishima Y, Wohlgemuth S, Liu B, Bower M, Carroll IM (2014). Induction of bacterial antigen-specific colitis by a simplified human microbiota consortium in gnotobiotic interleukin-10-/- mice. Infect Immun.

[94] Schäffler H, Herlemann DP, Alberts C, Kaschitzki A, Bodammer P, Bannert K (2016). Mucosa-attached bacterial community in Crohn's disease coheres with the clinical disease activity index. Environ Microbiol Rep.

[95] Laubitz D, Harrison CA, Midura-Kiela MT, Ramalingam R, Larmonier CB, Chase JH (2016). Reduced epithelial Na+/H+exchange drives gut microbial dysbiosis and promotes inflammatory response in T cell-mediated murine colitis. PLoS One 2016.

[96] Tchaptchet S, Fan TJ, Goeser L, Schoenborn A, Gulati AS, Sartor RB (2013). Inflammation-induced acid tolerance genes gadAB in luminal commensal *Escherichia coli* attenuate experimental colitis. Infection Immun.

[97] Darfeuille-Michaud A, Boudeau J, Bulois P, Neut C, Glasser AL, Barnich N (2004). High prevalence of adherent-invasive *Escherichia coli* associated with ileal mucosa in Crohn's disease. Gastroenterology.

[98] Liu Y, van Kruiningen HJ, West AB, Cartun RW, Cortot A, Colombel JF (1995). Immunocytochemical evidence of *Listeria*, *Escherichia coli*, and *Streptococcus antigens* in Crohn's disease. Gastroenterology.

[99] Atreya R, Neurath MF (2017). Current and future targets for mucosal healing in inflammatory bowel disease. Visceral Med.

[100] Hansen JJ, Sartor RB (2015). Therapeutic manipulation of the microbiome in IBD:Current results and future approaches. Curr Treatment Options Gastroenterol.

[101] Cénit MC, Matzaraki V, Tigchelaar EF, Zhernakova A (2014). Rapidly expanding knowledge on the role of the gut microbiome in health and disease. Biochim Biophys Acta.

[102] Kaser A, Zeissig S, Blumberg RS (2010). Inflammatory bowel disease. Annu Rev Immunol.

[103] Schreiber S, Dignass A, Peyrin-Biroulet L, Hather G, Demuth D, Mosli M (2018). Systematic review with meta-analysis:Real-world effectiveness and safety of vedolizumab in patients with inflammatory bowel disease. J Gastroenterol.

[104] Mosli MH, Rivera-Nieves J, Feagan BG (2014). T-cell trafficking and anti-adhesion strategies in inflammatory bowel disease:Current and future prospects. Drugs.

[105] Rutgeerts P, Van Assche G, Sandborn WJ, Wolf DC, Geboes K, Colombel JF (2012). Adalimumab induces and maintains mucosal healing in patients with Crohn's disease:Data from the EXTEND trial. Gastroenterology.

[106] D'Haens G, Sandborn WJ, Feagan BG, Geboes K, Hanauer SB, Irvine EJ (2007). A review of activity indices and efficacy end points for clinical trials of medical therapy in adults with ulcerative colitis. Gastroenterology.

[107] Kwapisz L, Mosli M, Chande N, Yan B, Beaton M, Micsko J (2015). Rapid fecal calprotectin testing to assess for endoscopic disease activity in inflammatory bowel disease:A diagnostic cohort study. Saudi J Gastroenterol.

[108] Malamut G, Afchain P, Verkarre V, Lecomte T, Amiot A, Damotte D (2009). Presentation and long-term follow-up of refractory celiac disease:comparison of Type I with Type II. Gastroenterology.

[109] Mosli MH, Feagan BG, Sandborn WJ, D'Haens G, Behling C, Kaplan K (2014). Histologic evaluation of ulcerative colitis:A systematic review of disease activity indices. Inflamm Bowel Dis.

[110] Mosli MH, Feagan BG, Zou G, Sandborn WJ, D'Haens G, Khanna R (2015). Reproducibility of histological assessments of disease activity in UC. Gut.

[111] Jairath V, Peyrin-Biroulet L, Zou G, Mosli M, Casteele NV, Pai RK (2019). Responsiveness of histological disease activity indices in ulcerative colitis:A post hoc analysis using data from the TOUCHSTONE randomised controlled trial. Gut.

[112] Brennan F, Maini R, Feldmann M (1992). TNFa-a pivotal role in rheumatoid arthritis?. Rheumatology.

[113] Yoshihara T, Shinzaki S, Kawai S, Fujii H, Iwatani S, Yamaguchi T (2017). Tissue drug concentrations of anti-tumor necrosis factor agents are associated with the long-term outcome of patients with Crohn's disease. Inflamm Bowel Dis.

[114] Poggi A, Benelli R, Venè R, Costa D, Ferrari N, Tosetti F (2019). Human gut-associated natural killer cells in health and disease. Front Immunol.

[115] Colombel JF, Rutgeerts P, Reinisch W, Esser D, Wang Y, Lang Y (2011). Early mucosal healing with infliximab is associated with improved long-term clinical outcomes in ulcerative colitis. Gastroenterology.

[116] Colombel JF, Sandborn WJ, Reinisch W, Mantzaris GJ, Kornbluth A, Rachmilewitz D (2010). Infliximab, azathioprine, or combination therapy for Crohn's disease. N Engl J Med.

[117] Atreya R, Zimmer M, Bartsch B, Waldner MJ, Atreya I, Neumann H (2011). Antibodies against tumor necrosis factor (TNF) induce T-cell apoptosis in patients with inflammatory bowel diseases via TNF receptor 2 and intestinal CD14(+) macrophages. Gastroenterology.

[118] Rutgeerts P, Sandborn WJ, Feagan BG, Reinisch W, Olson A, Johanns J (2005). Infliximab for induction and maintenance therapy for ulcerative colitis. N Engl J Med.

[119] Sandborn WJ, Colombel JF, D'Haens G, Van Assche G, Wolf D, Kron M (2013). One-year maintenance outcomes among patients with moderately-to-severely active ulcerative colitis who responded to induction therapy with adalimumab:Subgroup analyses from ULTRA 2. Aliment Pharmacol Ther.

[120] Magnusson MK, Strid H, Sapnara M, Lasson A, Bajor A, Ung KA (2016). Anti-TNF therapy response in patients with ulcerative colitis is associated with colonic antimicrobial peptide expression and microbiota composition. J Crohns Colitis.

[121] Varela E, Manichanh C, Gallart M, Torrejón A, Borruel N, Casellas F (2013). Colonisation by *Faecalibacterium prausnitzii* and maintenance of clinical remission in patients with ulcerative colitis. Aliment Pharmacol Ther.

[122] Li D, Yu G, Hu H, Li X, Wang Y, Xiao Y (2017). Characteristics of faecal microbiota in paediatric Crohn's disease and their dynamic changes during infliximab therapy. J Crohns Colitis.

[123] Ben-Horin S, Mao R, Chen M (2015). Optimizing biologic treatment in IBD:Objective measures, but when, how and how often?. BMC Gastroenterol.

[124] Knox NC, Forbes JD, Van Domselaar G, Bernstein CN (2019). The gut microbiome as a target for IBD treatment:Are we there yet?. Curr Treat Options Gastroenterol.

[125] Byndloss MX, Olsan EE, Rivera-Chávez F, Tiffany CR, Cevallos SA, Lokken KL (2017). Microbiota-activated PPAR-g signaling inhibits dysbiotic *Enterobacteriaceae* expansion. Science.

[126] Litvak Y, Byndloss MX, Tsolis RM, Bäumler AJ (2017). Dysbiotic *Proteobacteria* expansion:A microbial signature of epithelial dysfunction. Curr Opin Microbiol.

[127] Shaw KA, Bertha M, Hofmekler T, Chopra P, Vatanen T, Srivatsa A (2016). Dysbiosis, inflammation, and response to treatment:A longitudinal study of pediatric subjects with newly diagnosed inflammatory bowel disease. Genome Med.

[128] Machiels K, Sabino J, Vandermosten L, Joossens M, Arijs I, de Bruyn M (2017). Specific members of the predominant gut microbiota predict pouchitis following colectomy and IPAA in UC. Gut.

[129] Rajca S, Grondin V, Louis E, Vernier-Massouille G, Grimaud JC, Bouhnik Y (2014). Alterations in the intestinal microbiome (dysbiosis) as a predictor of relapse after infliximab withdrawal in Crohn's disease. Inflamm Bowel Dis.

[130] Moustafa A, Li W, Anderson EL, Wong EH, Dulai PS, Sandborn WJ (2018). Genetic risk, dysbiosis, and treatment stratification using host genome and gut microbiome in inflammatory bowel disease. Clin Transl Gastroenterol.

[131] Imhann F, Vich Vila A, Bonder MJ, Fu J, Gevers D, Visschedijk MC (2018). Interplay of host genetics and gut microbiota underlying the onset and clinical presentation of inflammatory bowel disease. Gut.

[132] Schroeder BO, Bäckhed F (2016). Signals from the gut microbiota to distant organs in physiology and disease. Nat Med.

[133] Guinane CM, Cotter PD (2013). Role of the gut microbiota in health and chronic gastrointestinal disease:Understanding a hidden metabolic organ. Ther Adv Gastroenterol.

[134] McIlroy J, Ianiro G, Mukhopadhya I, Hansen R, Hold GL (2018). Review article:The gut microbiome in inflammatory bowel disease-avenues for microbial management. Aliment Pharmacol Ther.

[135] Rehman A, Rausch P, Wang J, Skieceviciene J, Kiudelis G, Bhagalia K (2016). Geographical patterns of the standing and active human gut microbiome in health and IBD. Gut.

[136] Schenk M, Bouchon A, Birrer S, Colonna M, Mueller C (2005). Macrophages expressing triggering receptor expressed on myeloid cells-1 are underrepresented in the human intestine. J Immunol.

[137] Alipour M, Zaidi D, Valcheva R, Jovel J, Martínez I, Sergi C (2015). Mucosal barrier depletion and loss of bacterial diversity are primary abnormalities in paediatric ulcerative colitis. J Crohns Colitis.

[138] Shah R, Cope JL, Nagy-Szakal D, Dowd S, Versalovic J, Hollister EB (2016). Composition and function of the pediatric colonic mucosal microbiome in untreated patients with ulcerative colitis. Gut Microbes.

[139] Quince C, Ijaz UZ, Loman N, Eren AM, Saulnier D, Russell J (2015). Extensive modulation of the fecal metagenome in children with Crohn's disease during exclusive enteral nutrition. Am J Gastroenterol.

[140] Rajilić-Stojanović M, Shanahan F, Guarner F, de Vos WM (2013). Phylogenetic analysis of dysbiosis in ulcerative colitis during remission. Inflamm Bowel Dis.

[141] Kaakoush NO, Day AS, Huinao KD, Leach ST, Lemberg DA, Dowd SE (2012). Microbial dysbiosis in pediatric patients with Crohn's disease. J Clin Microbiol.

[142] Morgan XC, Tickle TL, Sokol H, Gevers D, Devaney KL, Ward DV (2012). Dysfunction of the intestinal microbiome in inflammatory bowel disease and treatment. Genome Biol.

